# Plumbagin Triggers Cuproptosis in Hepatocellular Carcinoma (HCC) via the DNA‐Methyltransferase 1 (DNMT1)/microRNA‐302a‐3p (miR‐302a‐3p)/ATPase Copper Transporting Beta (ATP7B) Axis

**DOI:** 10.1002/mco2.70312

**Published:** 2025-08-03

**Authors:** Chuyu Wang, Hao Wang, Chong Wang, Tongtong Tian, Anli Jin, Yu Liu, Ran Huo, Te Liu, Baishen Pan, Wei Guo, Wenjing Yang, Beili Wang

**Affiliations:** ^1^ Department of Laboratory Medicine Zhongshan Hospital Fudan University Shanghai China; ^2^ Shanghai Geriatric Institute of Chinese Medicine Shanghai University of Traditional Chinese Medicine Shanghai China; ^3^ Department of Laboratory Medicine Shanghai Geriatric Medical Center Shanghai China; ^4^ Department of Laboratory Medicine Wusong Central Hospital Shanghai China; ^5^ Department of Laboratory Medicine Xiamen Branch Zhongshan Hospital Fudan University Xiamen China

**Keywords:** ATPase copper transporting beta (ATP7B), cuproptosis, DNA‐methyltransferase 1 (DNMT1), hepatocellular carcinoma (HCC), plumbagin (PLB)

## Abstract

Induction of cuproptosis in tumor cells is an emerging direction for cancer drug development. Plumbagin (PLB), a natural biological molecule, has anticancer activities, partially via copper‐dependent mechanisms. But it remains unclear if PLB can induce cuproptosis in hepatocellular carcinoma (HCC). In this study, PLB showed HCC‐suppressive activities and caused representative molecular phenotypes of cuproptosis, whereas tetrathiomolybdate, an inhibitor of cuproptosis, could alleviate these effects the most. The mRNA and protein expression levels of the primary hepatic copper exporter, ATPase copper transporting beta (ATP7B), decreased in PLB‐treated HCC cells, which might cause the accumulation of intracellular copper and trigger cuproptosis. An upstream ATP7B‐regulatory microRNA, microRNA‐302a‐3p (miR‐302a‐3p), was identified by quantification and validated by the overexpression/inhibition experiment and luciferase reporter assay. Moreover, PLB was found to reduce the protein level of DNA‐methyltransferase 1 (DNMT1), thereby enhancing the promoter hypomethylation and the expression of miR‐302a‐3p. Gene manipulation experiments further demonstrated that ATP7B, miR‐302a‐3p, and DNMT1 mediated PLB‐induced cuproptosis. Preliminary clinical analyses showed that low ATP7B expression levels were associated with better prognosis, supporting the importance of ATP7B‐lowering therapeutic strategies in HCC. Together, our results indicate that PLB triggers HCC cuproptosis via the DNMT1/miR‐302a‐3p/ATP7B axis, providing a potential therapeutic strategy for HCC.

## Graphical abstract

A natural biological molecule, plumbagin (PLB), suppresses hepatocellular carcinoma via cuproptosis. PLB treatment transcriptionally downregulates the primary hepatic copper exporter, ATP7B, thereby accumulating intracellular copper ions to trigger cuproptosis. ATP7B downregulation by PLB is mediated by the DNMT1/miR‐302a‐3p axis through epigenetic processes

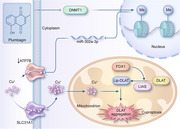

## 1 Introduction

Globally, liver cancer ranks sixth in terms of incidence among all types of cancer, and its predominant form, hepatocellular carcinoma (HCC), occupies nearly 80%–90% of the primary cases [1]. Yearly, about 725,000 people are newly diagnosed with HCC, and 664,000 people die of HCC [2, 3]. Radical resection remains the preferred curative treatment for patients with early‐stage HCC [4, 5]. But more than 60% of the newly diagnosed cases are at an intermediate or advanced stage [6]. Among these patients, the ones with local disease could receive liver‐directed therapies, including transcatheter arterial chemoembolization (TACE) and radiation [3]. Nevertheless, HCC patients receiving any of these therapies still have a considerable risk of recurrence [3, 7]. Therefore, drug therapy is a significant complement. On the one hand, it could be added to improve the outcomes of resection, TACE, and radiation [4, 5, 7]. On the other hand, it helps to control or reduce the size of tumors in intermediate and advanced patients, even allowing some of them to receive radical resection [4, 5, 7]. The mechanisms of drug action are distinct, causing different modalities of cell death. Sorafenib may induce ferroptosis of HCC cells via the inhibition of RAF and HBXIP/SCD axis; 5‐fluorouracil causes apoptosis by damaging DNA, whereas programmed cell death‐1 blockade enhances immune attack from T cells [8, 9, 10]. Although great progress has been made in drug development, the response rate using the combination of target therapy and immunotherapy drugs is less than 20% in advanced patients, and the 5‐year overall survival rate of HCC patients is low, remaining a global health challenge [11]. Hence, novel therapeutic targets and new modalities of cell death are always worth investigating.

Cuproptosis is a newly defined modality of cell death. It was described in detail for the first time by Tsvetkov et al. in 2022 [12]. The accumulation of Cu in mitochondria triggers cuproptosis by promoting the aggregation of lipoylated dihydrolipoamide S‐acetyltransferase (DLAT), ultimately resulting in proteotoxic stress and cell death [13]. The identified key regulators of cuproptosis include proteins in the lipoic acid pathway and in the pyruvate dehydrogenase (PDH) complex [12, 13, 14, 15]. Ferredoxin‐1 (FDX1), lipoic acid synthase (LIAS), lipoyltransferase 1 (LIPT1), and dihydrolipoamide dehydrogenase (DLD) in the lipoic acid pathway, as well as dihydrolipoamide S‐acetyltransferase (DLAT), pyruvate dehydrogenase E1 subunit alpha 1 (PDHA1), and pyruvate dehydrogenase E1 subunit beta (PDHB) in the PDH complex, are believed to promote cuproptosis, whereas metal regulatory transcription factor 1 (MTF1), glutaminase (GLS), and cyclin‐dependent kinase inhibitor 2A (CDKN2A) in the PDH complex inhibit cuproptosis [12, 14]. Meanwhile, several proteins, including METTL16 and MELK, have been reported to regulate cuproptosis via the key regulators [12, 14]. Elesclomol, acting as a copper ionophore, is the first compound reported to induce cuproptosis in lung cancer cells [16]. In colorectal cancer cells, inhibition of aerobic glycolysis by 4‐octyl itaconate was found to promote cuproptosis [17]. Treatment nanosystems are another approach to induce cuproptosis. The Au NCs‐Cu^2+^@SA‐HA core‐shell nanohybrid gels developed by Yang et al. could deliver Cu^2+^ to the tumor, triggering cuproptosis [18]. These studies indicate that the drug‐induced cuproptosis of tumor cells is a promising direction for HCC therapies.

The development of drugs largely depends on the existing drug molecule reserve. Synthetic small‐molecule drug library is a common source of drug reserve, while natural biological molecules, such as natural polyphenolic compounds, ursolic acid derivatives, anisomycin, and stigmasterol, also play an important role [19, 20, 21, 22]. Plumbagin (PLB), a quinoid compound derived from the root of *Plumbago zeylanica L*., has shown anticancer activity both in vitro and in vivo against breast, skin, lung, and liver cancers [23]. It could suppress HCC by inducing apoptosis and autophagy [24, 25]. There are also reports showing that PLB induces copper‐related death in cancer cells [26, 27]. In skin cancer cells, PLB induced cell death via a copper‐mediated redox cycle mechanism [26]. In addition, the complexation of PLB with copper significantly increases reactive oxygen species (ROS) in skin cancer cells and breast cancer cells, showing a stronger activity than cisplatin [26, 27]. However, whether PLB can induce cuproptosis in tumor cells, especially in HCC cells, remains unclear.

Epigenetic processes influence the flow of information between constant DNA sequences and variable patterns of gene expression. Unlike irreversible genetic alterations, the reversible nature of epigenetic processes has led to widespread involvement of epigenetic mechanisms in drug actions [28]. For example, drug‐induced microRNAs (miRNAs) could bind to the 3′ untranslated region (UTR) target sites within mRNA transcripts to achieve post‐transcriptional gene silencing [29]. Some other drugs may alter the regulatory enzymes of DNA methylation, thereby causing transcriptional and expressional changes of genes or miRNAs [28, 30]. Therefore, the epigenetic machinery may also be involved in the action of PLB on HCC cells.

In this study, we conducted both in vitro and in vivo experiments to explore the biological impact of PLB on HCC cells and assess its potential to induce cuproptosis‐related molecular phenotypes. Furthermore, we explored the epigenetic mechanism by which PLB triggers cuproptosis in HCC cells. Our findings may provide new insights into the mechanism of cuproptosis and the anticancer effects of PLB.

## 2 Results

### 2.1 HCC Cell Growth Can be Suppressed by PLB and Rescued by Tetrathiomolybdate

**FIGURE 1:**
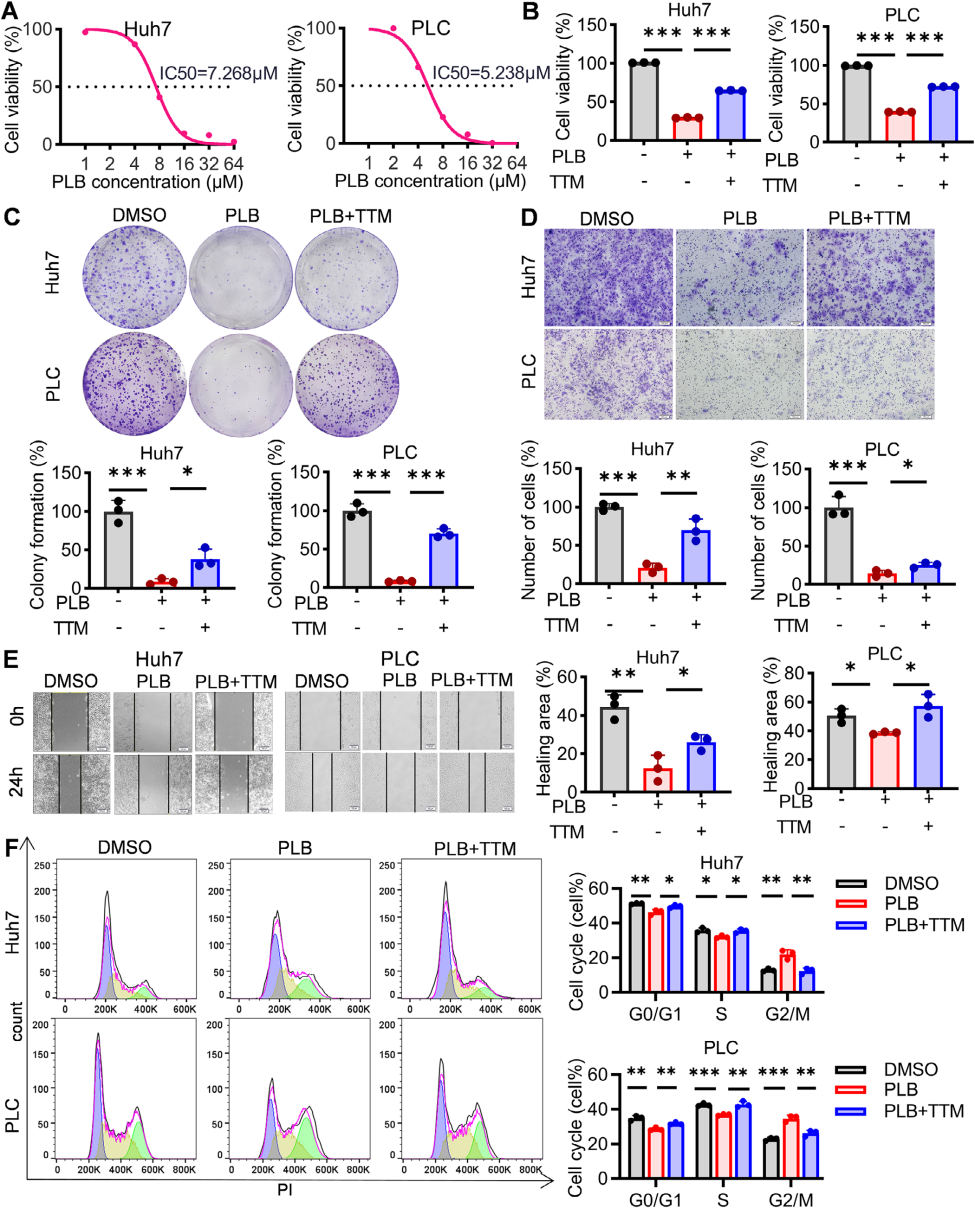
PLB inhibits the cell growth, proliferation, and migration of HCC cells. (A) Dose‐course effects of PLB on the viability of HCC cells. IC50 for either cell line was labeled. *N* = 5. (B) The effect of TTM on the viability of HCC cells under PLB treatment (6 µM). Colony formation (C), Transwell (D), wound‐healing (E), and cell cycle analysis (F) assays of HCC cells under PLB treatment. TTM was used to rescue cells from PLB treatment. The colors purple, yellow, and green represent the G0/G1, S, and G2/M phases, respectively. *N* = 3; ^∗^ *p* < 0.05, ^∗∗^ *p* < 0.01, and ^∗∗∗^ *p* < 0.001.

First, we tested the effect of PLB on cell viability in two human‐derived HCC cell lines (Huh7 and PLC cells). In the 24‐h PLB treatment experiment, PLB inhibited the proliferation of HCC cells in a dose‐dependent manner, and at the inhibition rate of 50%, its concentrations were 7.3 µM for Huh7 cells and 5.2 µM for PLC cells, respectively (Figure 1A). Meanwhile, tetrathiomolybdate (TTM), a chelator of copper that could inhibit cuproptosis, attenuated PLB‐induced reduction of cell viability, and its effect was stronger than ferroptosis, necroptosis, and apoptosis inhibitors, suggesting that PLB suppressed the growth of HCC cells mainly via cuproptosis (Figure 1B and Figure S1A). Further analyses showed that the inhibitory effect of PLB on the viability of HCC cells was also time‐dependent (Figure S1B).

Similarly, PLB reduced the number of colonies formed, decreased the number of cells migrating through the membrane, and impaired HCC cells’ ability to heal the wound, whereas TTM somewhat alleviated the effects of PLB, further supporting that PLB might suppress HCC by triggering cuproptosis (Figure 1C–E). In addition, compared to dimethyl sulfoxide (DMSO) and PLB plus TTM groups, the PLB group had a higher cell percentage at the G2/M stage, indicating that PLB‐induced cuproptosis caused G2/M cell cycle arrest of HCC cells (Figure 1F).

### 2.2 PLB Induces Changes in Cuproptosis‐Associated RNAs and Proteins

**FIGURE 2:**
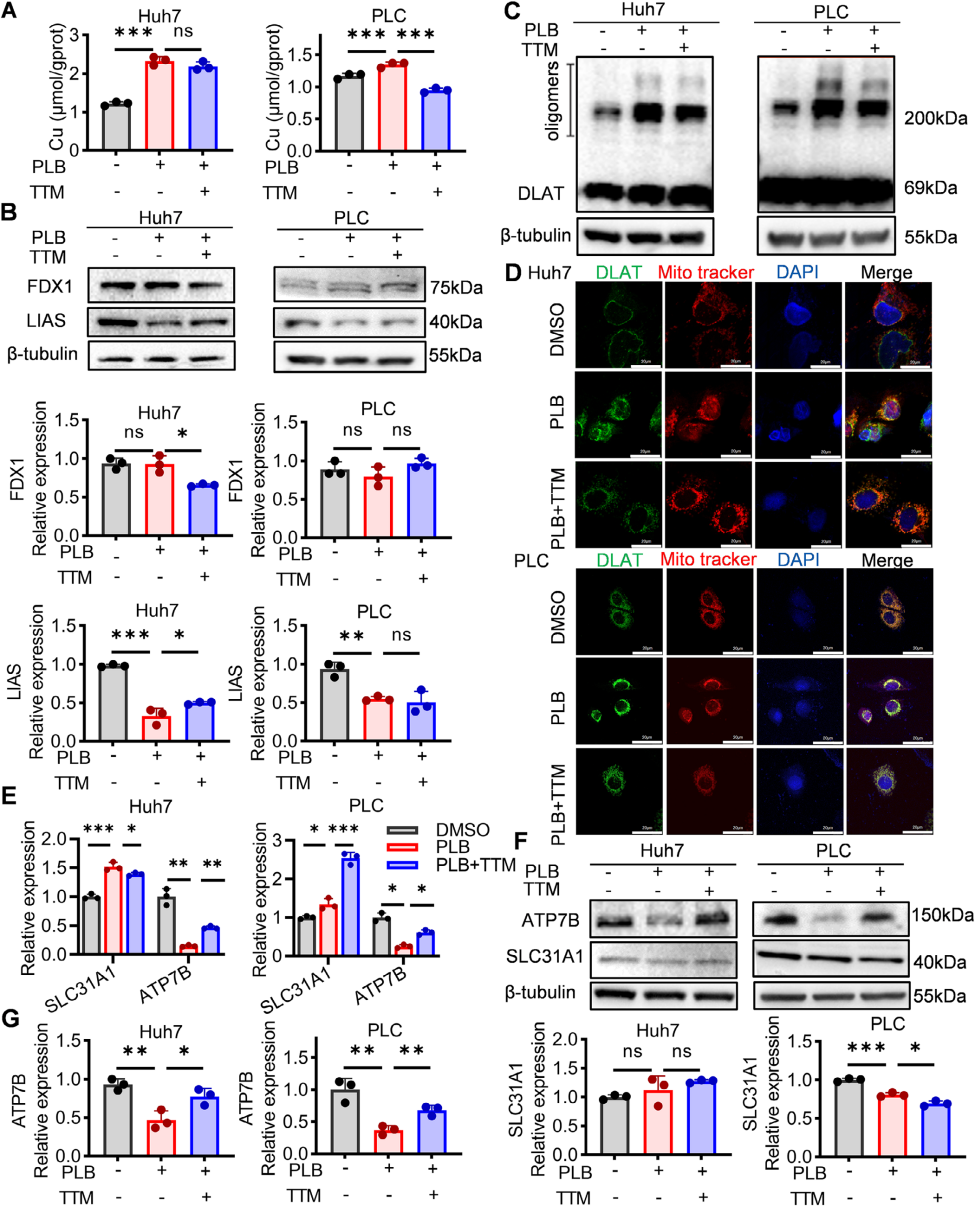
PLB induces cuproptosis‐associated molecular phenotypes in HCC cells. (A) Intracellular copper ion concentration after PLB treatment (6 µM). (B) Western blotting analysis of FDX1 and LIAS in PLB‐treated HCC cells. DLAT protein oligomerization was analyzed by Western blotting (C) and immunofluorescence (D). DLAT—green, Mitotracker—red, DAPI—blue. Scale bar: 20 µm. The mRNA (E) and protein (F and G) levels of ATP7B and SLC31A1 in PLB‐treated HCC cells. *N* = 3; ^∗^ *p* < 0.05, ^∗∗^ *p* < 0.01, and ^∗∗∗^ *p* < 0.001; ns, not significant.

To further characterize PLB‐induced cuproptosis [31], we measured the concentration of intracellular copper ions and the expression of essential cuproptosis‐related proteins, including FDX1, LIAS, and DLAT, in HCC cells. PLB treatment increased the intracellular copper concentration, indicating that PLB caused the accumulation of copper ions in HCC cells (Figure 2A). One of the Fe‐S cluster proteins, LIAS, showed reduced expression on Western blots under PLB treatment (Figure 2B). Moreover, the increased oligomers of DLAT on Western blots, as well as the enhanced overlap between DLAT and mitochondria in immunofluorescence, reveal that PLB promoted DLAT aggregation (Figure 2C,D). All these results together support that PLB triggered cuproptosis in HCC cells.

In hepatocyte‐derived HCC cells, the homeostasis of intracellular copper ions is regulated by the copper importer, solute carrier family 31 member 1 (SLC31A1), and the primary hepatic exporter, ATPase copper transporting beta (ATP7B) [12, 31]. Thus, the mRNA and protein levels of both SLC31A1 and ATP7B were examined. PLB consistently reduced ATP7B mRNA levels and protein levels in both Huh7 and PLC cells, whereas its effects on SLC31A1 were not consistent between mRNA and protein, or between Huh7 and PLC cells (Figure 2E–G). Meanwhile, TTM might restore ATP7B mRNA levels and protein levels from the reduction induced by PLB (Figure 2E–G). These results suggest that PLB treatment led to a transcriptional downregulation of ATP7B.

### 2.3 PLB Enhances Oxidative Stress and Mitochondrial Dysfunction in HCC Cells

**FIGURE 3:**
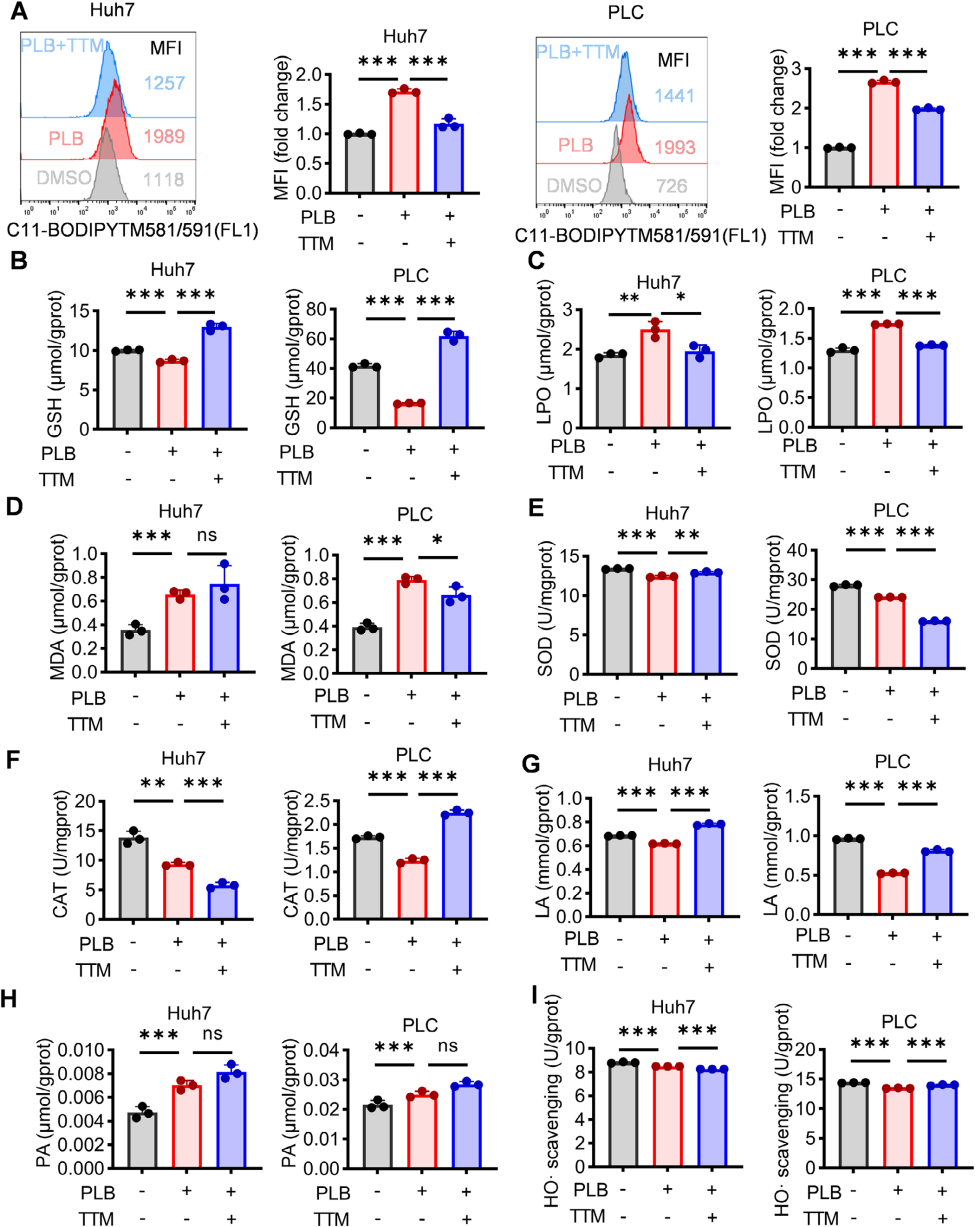
PLB increases oxidative stress in HCC cells. (A) The accumulation of lipid‐ROS was detected in PLB‐treated HCC cells (6 µM). (B–H) The content of intracellular GSH, LPO, MDA, SOD, CAT, LA, and PA of HCC cells after PLB treatment. (I) The hydroxyl radical (HO·) scavenging activity of HCC cells after PLB treatment. *N* = 3; ^∗^ *p* < 0.05, ^∗∗^ *p* < 0.01, and ^∗∗∗^ *p* < 0.001; ns, not significant.

Generation of ROS is a commonly reported phenomenon in copper‐induced cell death. Copper‐induced ROS also increases lipid peroxidation and depletes glutathione (GSH), making cells more susceptible to oxidative damage [32]. We analyzed oxidative stress in PLB‐treated HCC cells. PLB significantly increased the mean fluorescence intensity (MFI) of C11‐BODIPY staining in Huh7 and PLC cells, whereas the MFI decreased with TTM added, indicating PLB induced the accumulation of lipid‐ROS (Figure 3A). The depletion of GSH was also detected under PLB treatment, and the depletion effect was eliminated by TTM (Figure 3B). Meanwhile, the measurements of metabolite concentration and enzyme activity showed that intracellular peroxides, including lipid hydroperoxide (LPO) and malondialdehyde (MDA), increased, and the activities of antioxidant enzymes, such as superoxide dismutase (SOD) and catalase (CAT), declined in PLB‐treated HCC cells (Figure 3C–F). All these findings, as well as the reduced lactate acid (LA), increased pyruvic acid (PA), and weakened hydroxyl radical scavenging activity, support that PLB induced oxidative stress in HCC cells (Figure 3G–I). Furthermore, copper‐induced cellular oxidative stress can lead to mitochondrial dysfunction [31]. Transmission electron microscopy showed mitochondrial shrinkage, reduced cristae, increased membrane density, or swelling in HCC cells after PLB treatment (Figure S2). Hence, the molecular and morphological phenotypes of copper‐induced cell death were observed in PLB‐treated HCC cells, which were consistent with the finding that PLB triggered cuproptosis in HCC cells.

### 2.4 PLB Triggers Cuproptosis in HCC Cells via the DNMT1/MicroRNA‐302a‐3p (miR‐302a‐3p)/ATP7B Axis

**FIGURE 4:**
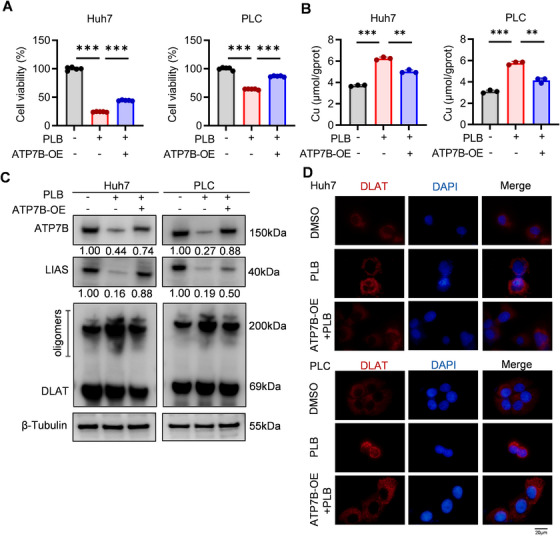
ATP7B mediates PLB‐induced cuproptosis. (A) Viability of ATP7B‐overexpressing (ATP7B‐OE) cells under PLB treatment (6 µM). *N* = 5. (B) Intracellular copper ion concentration of ATP7B‐OE cells after PLB treatment. *N* = 3. (C) Western blotting analysis of ATP7B, LIAS, and DLAT in PLB‐treated ATP7B‐OE cells. (D) Immunofluorescence for DLAT protein. DLAT—red, DAPI—blue. Scale bar: 20 µm. ^∗∗^ *p* < 0.01 and ^∗∗∗^ *p* < 0.001.

ATP7B, the exporter of copper, was shown to be transcriptionally downregulated by PLB, which probably accumulated intracellular copper ions and triggered cuproptosis. We overexpressed ATP7B in HCC cells to verify this hypothesis. Overexpression of ATP7B increased the viability and reduced intracellular copper concentration of PLB‐treated HCC cells (Figure 4A,B). With the reduction of copper, the consumption of Fe‐S cluster proteins (LIAS) and aggregation of DLAT, as well as cellular oxidative stress, were also alleviated (Figure 4C,D and Figure S3). These findings support that ATP7B is a key factor in PLB‐induced cuproptosis.

**FIGURE 5:**
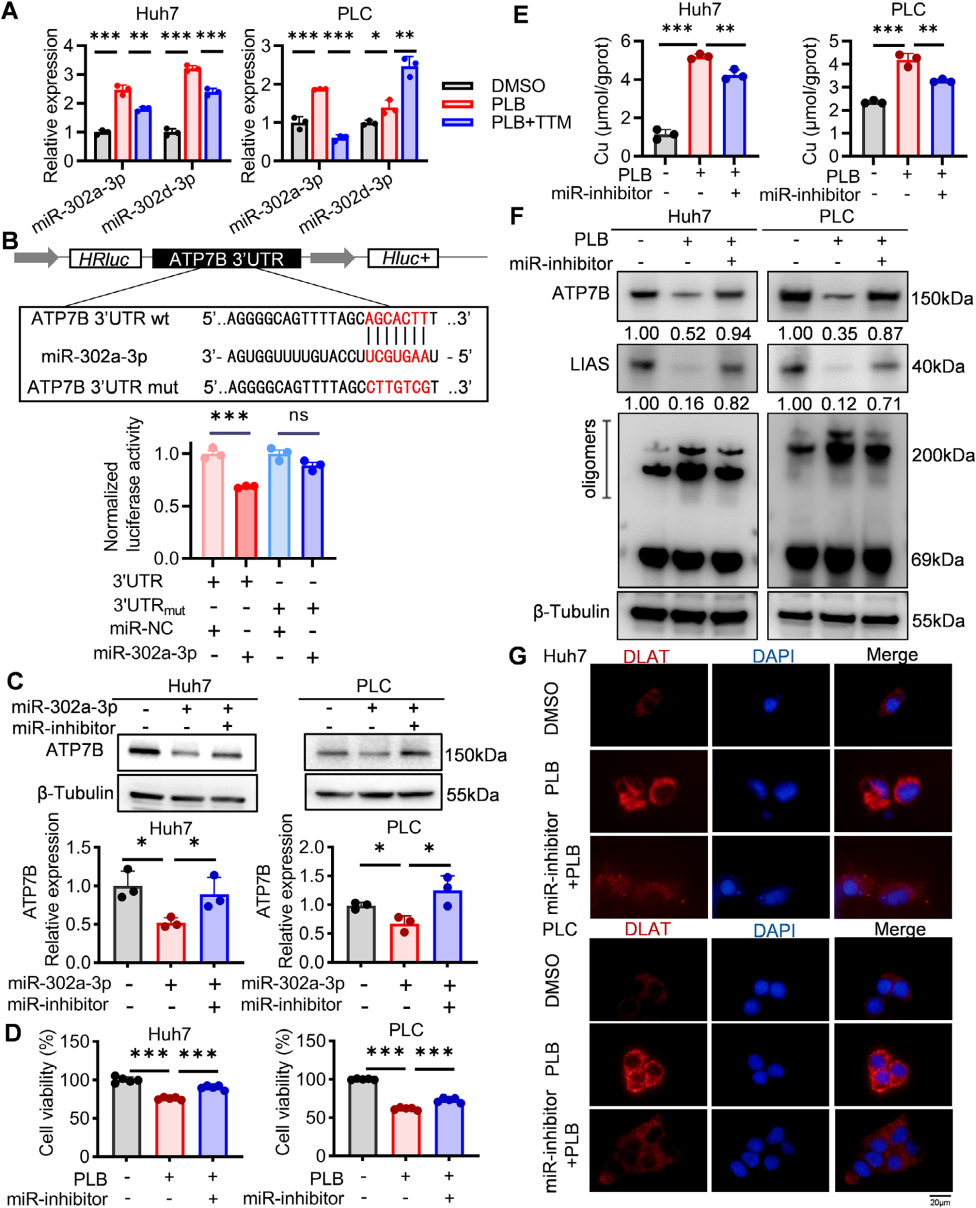
miR‐302a‐3p mediates PLB‐induced cuproptosis by regulating ATP7B. (A) The expression levels of miR‐302a‐3p and miR‐302d‐3p in HCC cells after PLB treatment (6 µM). *N* = 3. (B) Activities of luciferase report vectors containing wild‐type (wt) or mutated (mut) ATP7B 3’UTR sequence. HRluc and Hluc^+^ represent Renilla luciferase gene and firefly luciferase gene, respectively. *N* = 3. (C) The protein expression level of ATP7B in HCC cells with miR‐302a‐3p overexpression or inhibition. *N* = 3. (D) Viability of miR‐302a‐3p‐inhibiting cells under PLB treatment. *N* = 5. (E) Intracellular copper ion concentration of miR‐302a‐3p‐inhibiting cells after PLB treatment. *N* = 3. (F) Western blotting analysis of ATP7B, LIAS, and DLAT in PLB‐treated miR‐302a‐3p‐inhibiting cells. (G) Immunofluorescence for DLAT protein. DLAT—red, DAPI—blue. Scale bar: 20 µm. ^∗^ *p* < 0.05, ^∗∗^ *p* < 0.01, and ^∗∗∗^ *p* < 0.001; ns, not significant.

In epigenetics, miRNAs play an important role in drug‐induced gene silencing. We screened the miRNAs potentially regulating ATP7B from online databases and examined their expression in PLB‐treated HCC cells. Fifteen miRNAs with the potential to silence ATP7B were identified and two of them, miR‐302a‐3p and miR‐302d‐3p, were increased by PLB treatment (Figures 5A and S4A,B). However, the transfection of miR‐302d‐3p could not consistently reduce ATP7B protein levels in HCC cells (Figure S4C). The ATP7B‐regulating function of miR‐302a‐3p was further examined in a dual‐luciferase reporter assay and an inhibition experiment. Overexpression of miR‐302a‐3p impaired the activity of the luciferase carrying the binding site of miR‐302a‐3p from ATP7B 3’UTR, but it did not alter the activity of the luciferase with a mutated binding site (Figure 5B). The miR‐302a‐3p‐induced reduction of ATP7B protein level was attenuated by the miR‐302a‐3p inhibitor (Figure 5C). These results indicate that upregulated miR‐302a‐3p under PLB treatment might silence ATP7B by site‐specific binding to its 3’UTR. In PLB‐treated HCC cells, inhibition of miR‐302a‐3p could restore ATP7B proteins and prevent the accumulation of copper, thereby resisting cuproptosis and oxidative stress (Figures 5D–G and S5). It further supports that miR‐302a‐3p mediated PLB‐induced cuproptosis by regulating ATP7B.

**FIGURE 6:**
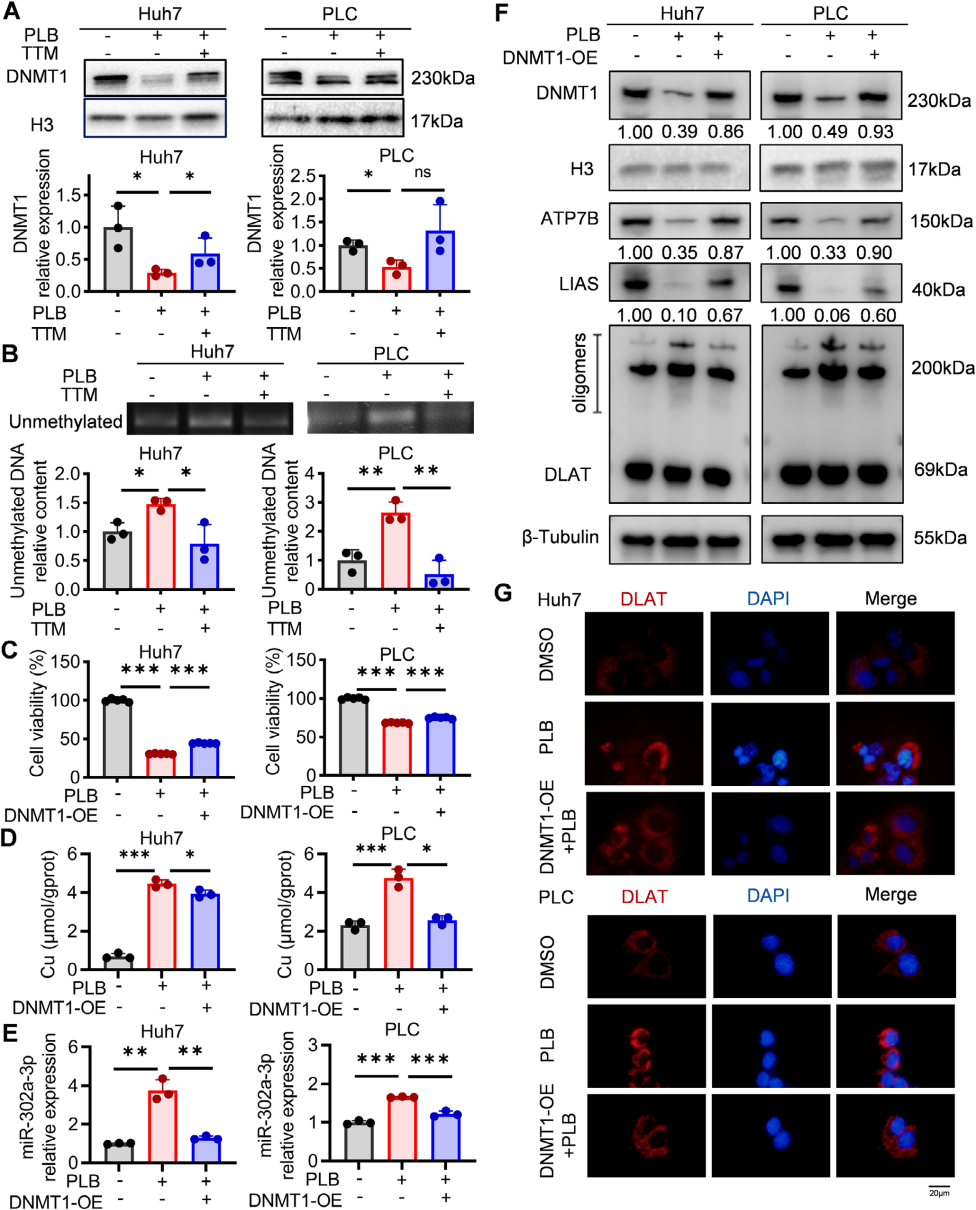
DNMT1 mediates PLB‐induced cuproptosis by regulating miR‐302a‐3p. (A) Western blotting analysis of DNMT1 in HCC cells after PLB treatment (6 µM). *N* = 3. (B) The unmethylation status of the miR‐302a‐3p promoter analyzed by methylation‐specific PCR. *N* = 3. (C) Viability of DNMT1‐overexpressing (DNMT1‐OE) cells under PLB treatment. *N* = 5. (D) Intracellular copper ion concentration of DNMT1‐OE cells after PLB treatment. *N* = 3. (E) The expression levels of miR‐302a‐3p in PLB‐treated DNMT1‐OE cells. *N* = 3. (F) Western blotting analysis of ATP7B, LIAS, and DLAT in PLB‐treated DNMT1‐OE cells. (G) Immunofluorescence for DLAT protein. DLAT—red, DAPI—blue. Scale bar: 20 µm. ^∗^ *p* < 0.05, ^∗∗^ *p* < 0.01, and ^∗∗∗^ *p* < 0.001; ns, not significant.

Methylation status of the promoter region epigenetically influences the transcriptional efficiency of miRNAs. We analyzed the protein expression of DNMT1, a key methyltransferase in HCC cells, and the promoter methylation of miR‐302a‐3p. The results showed that PLB reduced the protein level of DNMT1 and increased the amount of unmethylated promoter DNA, suggesting that PLB‐induced reduction of DNMT1 switched the promoter region of miR‐302a‐3p to an unmethylated status, thereby promoting the transcription of miR‐302a‐3p (Figure 6A,B). By overexpressing DNMT1, the viability of PLB‐treated HCC cells was improved (Figure 6C). Moreover, DNMT1 overexpression increased ATP7B protein level and reduced intracellular copper ions, as well as unmethylated promoter DNA of miR‐302a‐3p and miR‐302a‐3p itself (Figure 6D–F and Figure S6F). As a result, cuproptosis‐related molecular phenotypes and oxidative stress were partly eliminated, demonstrating that PLB‐induced cuproptosis was mediated by the DNMT1/miR‐302a‐3p/ATP7B axis (Figure 6F,G and Figure S6A–E).

### 2.5 PLB Suppresses Tumor Growth, Induces Cuproptosis, and Enhances Oxidative Stress in Vivo

**FIGURE 7:**
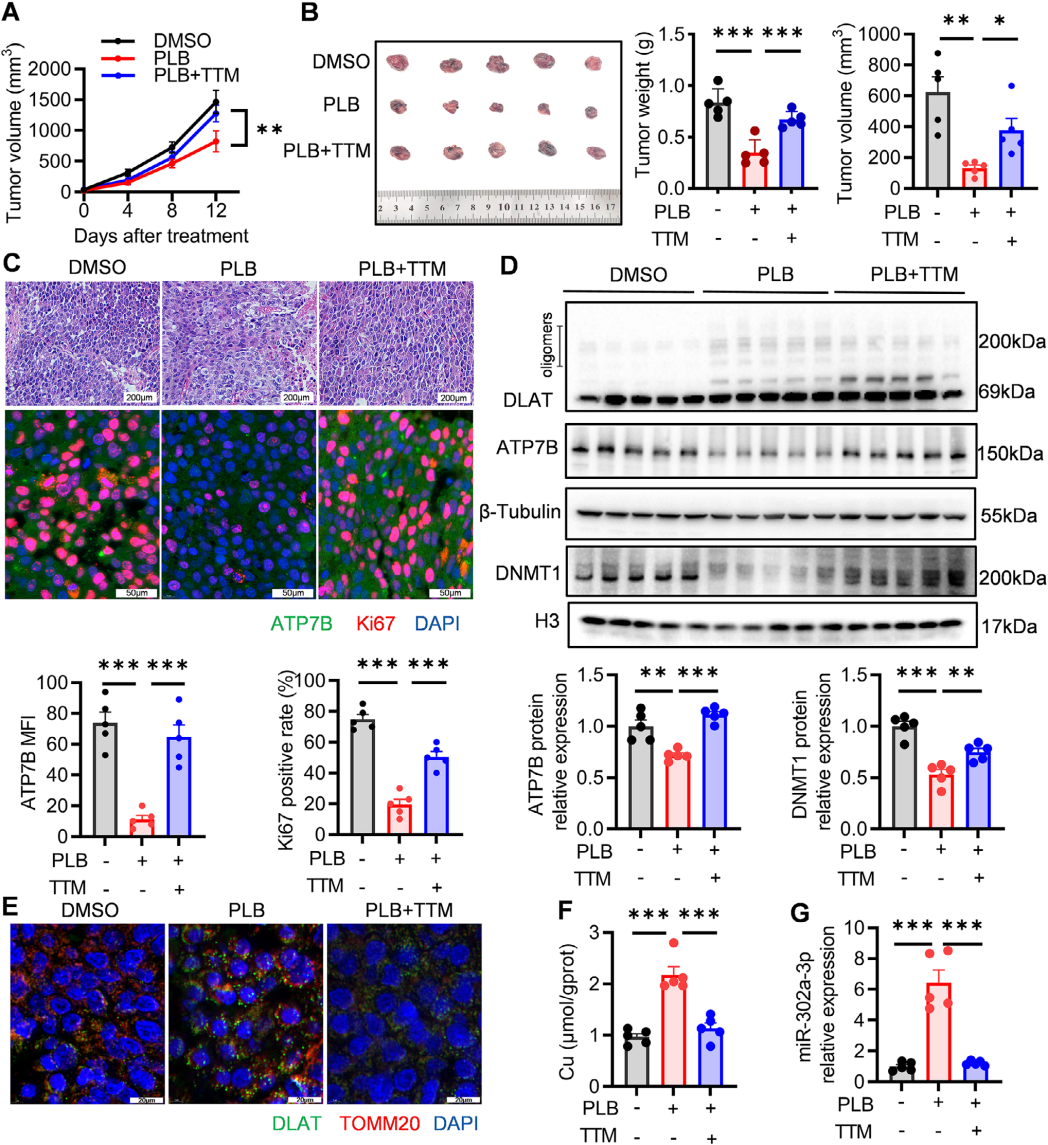
PLB suppresses tumor growth and induces cuproptosis in vivo. (A) The growth curve of xenograft tumors. (B) Sizes and weights of xenograft tumors. (C) Representative images of tumor tissues. Images on the top are H&E‐stained. Scale bars: 200 µm. Images on the bottom are co‐stained for ATP7B and Ki67. ATP7B—green, Ki67—red, DAPI—blue. Scale bars: 50 µm. ATP7B MFI and Ki67 positive rate were quantified. (D) Western blotting analysis of ATP7B, DNMT1 protein expression, and DLAT oligomerization in tumor tissues. (E) Representative immunofluorescent co‐staining images for DLAT and TOMM20, a mitochondrial marker. DLAT—green, TOMM20—red, DAPI—blue. Scale bar: 20 µm. (F) Copper ion concentration of tumor tissues. (G) The expression levels of miR‐302a‐3p in tumor tissues. *N* = 5; ^∗^ *p* < 0.05, ^∗∗^ *p* < 0.01, and ^***^ *p* < 0.001.

Next, we assessed the in vivo anticancer activity of PLB in an orthotopic xenograft mouse model. A tolerated dose of PLB, which was reported in previous studies [24] and validated by us (Figure S7), was administered to mice. The volumes of tumors from the PLB group were smaller than those from the DMSO group and the PLB plus TTM group, showing the in vivo tumor‐suppressing effects of PLB‐induced cuproptosis (Figure 7A,B). In hematoxylin and eosin (H&E) staining, the tumor cells were loosely distributed after PLB treatment, whereas the distribution of DMSO or PLB plus TTM‐treated tumor cells was tight (Figure 7C). Meanwhile, tumors from PLB‐treated mice had a lighter fluorescence intensity of ATP7B and fewer Ki67‐positive cells in immunofluorescence staining (Figure 7C). Together, these staining results indicate that PLB could reduce ATP7B expression and kill HCC cells in vivo.

The molecular phenotypes of cuproptosis were examined in tumors from PLB‐treated mice. With the reduction of ATP7B, increased intracellular copper ions, increased oligomers, and enhanced staining of DLAT were detected in PLB‐treated tumors, showing that PLB triggered HCC cuproptosis in vivo (Figure 7D–F). The reduced DNMT1 protein levels, increased unmethylated promoter amounts, increased miR‐302a‐3p levels, and reduced ATP7B mRNA levels in PLB‐treated tumor samples further support that the DNMT1/miR‐302a‐3p/ATP7B axis mediated cuproptosis triggered by PLB in vivo (Figure 7D,G and Figure S8I,J). Parameters related to oxidative stress, including GSH, LPO, MDA, SOD, CAT, LA, PA, and hydroxyl radical scavenging activity, were measured using tumor samples. Their changing patterns under PLB treatment were similar to those in the in vitro experiments, indicating that PLB enhanced oxidative stress in tumors (Figure S8A–H).

### 2.6 Low Expression of ATP7B in HCC Is Somewhat Associated With Better Outcomes

**FIGURE 8:**
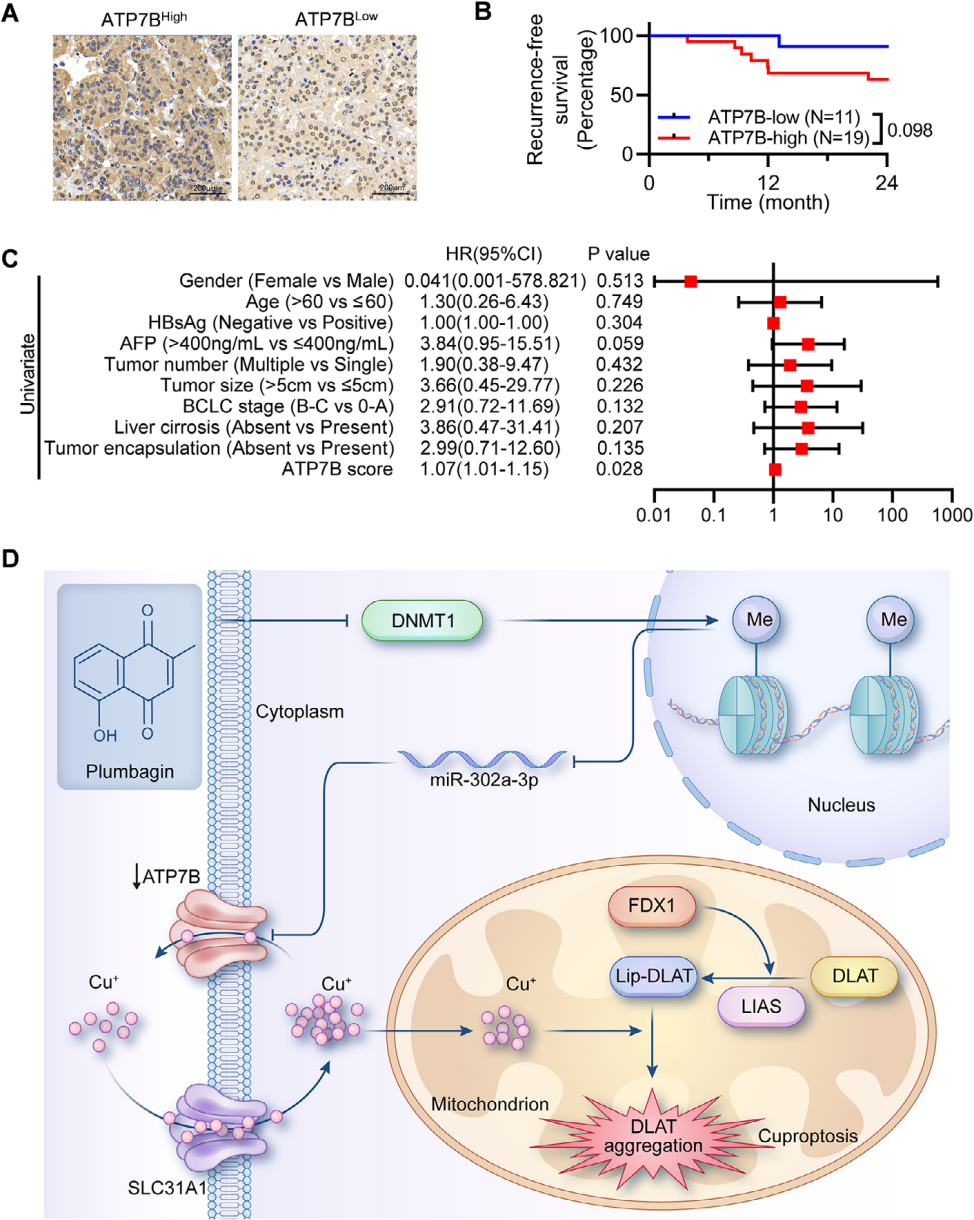
Prognostic value of ATP7B in HCC. (A) ATP7B‐staining HCC tissue samples. Representative images for both ATP7B‐high and ATP7B‐low immunohistochemistry samples are shown. (B) Prognostic analysis of ATP7B using clinical HCC tissue samples. (C) Univariate COX regression analysis. (D) Schematic diagram of PLB‐induced cuproptosis in HCC cells.

To further explore the influence of ATP7B expression on patient prognosis, we analyzed the recurrence‐free survival of HCC patients using ATP7B‐stained tumor samples. The ATP7B expression was scored, and the optimal threshold of ATP7B was determined (Figure 8A). The patients with low ATP7B expression levels had longer recurrence‐free survival with a *p*‐value of 0.098 (Figure 8B). A univariate COX regression analysis showed that the ATP7B score was a risk factor for recurrence (Figure 8C).

In summary, our study showed that PLB treatment could reduce the DNMT1 protein level in HCC cells (Figure 8D). The reduction of DNMT1 switched the promoter region of miR‐302a‐3p to an unmethylated status, thereby promoting the transcription of miR‐302a‐3p. Upregulated miR‐302a‐3p silenced ATP7B by site‐specific binding to its 3’UTR. The downregulation of ATP7B induced the accumulation of intracellular copper ions and finally triggered cuproptosis (Figure 8D). PLB may play an anticancer role by itself or form a complex with copper (Figure S9A). It is possible that PLB itself or the PLB‐copper complex directly targets DNMT1, resulting in the degradation of DNMT1. The binding affinity data from molecular docking analyses supported this hypothesis (Figure S9B). Furthermore, the stronger binding affinity of the PLB‐copper complex indicates that the complex was more likely to be the active form in PLB‐induced cuproptosis (Figure S9B,C). In addition to chelate‐free copper ions, TTM probably competed with PLB to chelate copper ions in the PLB‐copper complex and broke the complex, which may explain why TTM also affected upstream molecules, including DNMT1, miR‐302a‐3p, and ATP7B (Figure 8D and Figure S9).

## 3 Discussion

Since the first description of cuproptosis, a lot of efforts have been made to assess the value of cuproptosis in the management of HCC [33]. At the beginning, people focused on the expression level and prognostic value of cuproptosis‐related molecules, including mRNAs, miRNAs, long non‐coding RNAs (lncRNAs), and RNA methylation [34, 35, 36]. For example, a remarkable downregulation of FDX1, one of the key regulators of cuproptosis, was found in HCC, and the magnitude of its downregulation correlated with shortened survival. [34]. Recently, studies have shown that drug‐induced cuproptosis represents a promising therapeutic intervention strategy [37, 38, 39]. Yin et al. discovered that the LINC02362/hsa‐miR‐18a‐5p/FDX1 axis facilitates cuproptosis and enhances the sensitivity of HCC to oxaliplatin [37]. Liang et al. uncovered that ARID1A loss increases dependence on the tricarboxylic acid cycle, sensitizing HCC cells to elesclomol, a cuproptosis‐inducing drug [38]. Zhang et al. demonstrated that the combination of disulfiram/Cu and *x*CT inhibition shows stronger suppression of HCC cells, as it activates both ferroptosis and cuproptosis [39]. Moreover, Yang et al. discovered a novel cuproptosis‐inducing molecule, LGOd1, that targets copper chaperone for superoxide dismutase for inhibition of HCC via deep learning [40]. In our study, we reported that a natural product, PLB, could trigger cuproptosis by downregulating the primary hepatic copper exporter, ATP7B, thereby suppressing HCC. To our knowledge, our study is the first report comprehensively characterizing the cuproptosis‐related molecular phenotypes, including elevated intracellular copper concentration, consumption of Fe‐S cluster proteins, aggregation of DLAT, and production of ROS, in drug‐induced cuproptosis of HCC, simultaneously in vitro and in vivo. Meanwhile, PLB is believed to be the first ATP7B‐targeting cuproptosis‐inducing natural biological molecule for HCC.

The epigenetic elements, miRNAs, are important players in gene silencing and widely investigated. In our study, we identified two candidate miRNAs possibly regulating ATP7B by quantifying the expression of potential ATP7B‐regulating miRNAs under PLB treatment, and validated their regulatory abilities through the overexpression/inhibition experiment and luciferase reporter assay. Finally, we concluded that miR‐302a‐3p mediates PLB‐induced downregulation of ATP7B. Our study is the first experimental evidence showing that ATP7B was regulated by miR‐302a‐3p. In previous studies, several other mechanisms, distinct from miRNA‐induced gene silencing, have been found to regulate ATP7B, including intracellular copper ions, post‐translational modifications, and transcriptional regulation. At the protein level, elevated copper levels increase ATP7B expression in the plasma membrane, and depletion of GSH or impaired GRX1‐mediated deglutathionylation hinders this increase [41, 42]. In addition, COMMD1 is believed to regulate the stability of newly synthesized ATP7B [43]. At the transcriptional level, 5‐fluorouracil is proven to significantly suppress ATP7B mRNA expression, while metal regulatory transcription factor 1 is a strong candidate for transcriptional regulators for ATP7B by binding to metal‐responsive element e in the ATP7B promoter [44, 45]. Our findings from cell and animal models suggest that alteration of ATP7B via these mechanisms is worth studying in the development of therapeutic strategies for HCC. Moreover, the preliminary clinical analyses show that low ATP7B expression levels are associated with better prognosis, supporting the importance of ATP7B‐lowering therapeutic strategies and extending its value to prognosis.

MiR‐302a‐3p serves as a tumor suppressor for non‐small‐cell lung cancer and HCC, and its targets include ACAT1 and PRKACB [46, 47]. Our study adds a new experimentally validated target, ATP7B, for miR‐302a‐3p and extends the value of miR‐302a‐3p in cancer management. Meanwhile, LINC01016, Circ_0001665, and lncRNA DARS‐AS1 have been reported to act as miRNA “sponges” and regulate miR‐302a‐3p in endometrial cancer cells, vestibular schwannoma cells, and non‐small‐cell lung cancer cells, respectively [46, 48, 49]. Besides non‐coding RNAs, the levels of miRNAs are also affected by DNA methylation [50]. There have been some experimental studies showing the effects of promoter methylation on the expression of miRNAs in HCC. MiR‐125b is a tumor suppressor, but the promoter hypermethylation keeps it inactivated in HCC [51]. The promoter hypomethylation may upregulate the expression of miR‐106a, thereby contributing to the progression of HCC [52]. Moreover, the promoter methylation also mediates hepatitis B virus‐induced hepatocarcinogenesis. Tumor suppressor miR‐18b is inhibited by hepatitis B virus X protein (HBx) via the methylation of CpG islands in its promoter [53]. Our study first reports the regulatory effect of the promoter methylation on miR‐302a‐3p and reveals that the promoter methylation of miR‐302a‐3p plays an important role in maintaining copper homeostasis, and its change may induce cuproptosis.

In our experiments, PLB reduced the protein expression of DNMT1 in HCC, probably by directly binding to DNMT1 in a monomer form or a PLB‐copper complex. And the reduction of DNMT1 was associated with the promoter hypomethylation of miR‐302a‐3p. The investigation of DNMT1 in HCC was initiated as early as 2001 [54, 55]. The upregulation of DNMT1 was detected in noncancerous liver tissues showing chronic hepatitis or cirrhosis, and it was further upregulated in HCC tissues [54]. The following studies show that increased protein expression of DNMT1 is correlated with the malignant potential and poor prognosis of HCC [56, 57]. The increased DNMT1, which is induced by HBx, may reduce E‐cadherin expression by methylation‐mediated promoter inactivation [57, 58]. Hepatocyte growth factor, one of the essential growth factors in the HCC microenvironment, represses tumor suppressor genes via DNMT1‐mediated DNA hypermethylation [59]. Due to these roles in HCC, multiple studies have explored whether DNMT1 is a promising therapeutic target in HCC [60, 61, 62]. A clinically approved DNMT1 inhibitor, 5‐azacytidine, was reported to induce caspase activation and apoptosis in HCC [60]. Another interesting study assessed the therapeutic efficacy of a dual G9a histone‐methyltransferase and DNMT1 inhibitor in HCC, showing a synthetic lethal effect of independent G9a and DNMT1 pharmacological targeting [62]. Now, the induction of cuproptosis by targeting DNMT1 becomes a novel, valuable approach to curing HCC.

Our study also has some limitations. First, the cell model and orthotropic xenograft mouse model were used to examine tumor‐suppressive effects of PLB‐induced cuproptosis, but they do not completely represent complex biological processes in the body. The findings should be validated in more models before clinical application. Second, although we showed that ATP7B is a risk factor for recurrence and a potential biomarker in prognosis, the small cohort limits the generalizability of these findings. The prognostic value of ATP7B should be further examined in large cohorts. Furthermore, the ATP7B‐lowering cuproptosis‐inducing therapeutic potential of PLB should also be examined clinically. Third, drug action is a complicated process. In this study, we figured out the key role of the DNMT1/miR‐302a‐3p/ATP7B axis in PLB‐induced cuproptosis. But the potential contributions of other transcriptional regulators and signaling pathways are not clear. For example, other miRNAs altered by PLB and their roles were not identified. The form and the way in which PLB reduced the protein expression of DNMT1 were not fully investigated either. Therefore, more experiments should be performed for a better understanding and application of PLB‐induced cuproptosis.

## 4 Conclusion

In this study, we performed in vitro and in vivo experiments to investigate the biological effects of PLB on HCC cells and to examine whether PLB could induce cuproptosis‐related molecular phenotypes. Furthermore, we explored the epigenetic mechanism by which PLB triggers cuproptosis in HCC cells and evaluated the prognostic value of related genes. The findings of our study may help better understand the mechanism of cuproptosis and the action of PLB.

## 5 Materials and Methods

### 5.1 Cell Culture

Huh7 and PLC cell lines, originated from HCC tissues, were granted by the Liver Cancer Institute of Zhongshan Hospital, Fudan University (China) and cultured at 37°C in Dulbecco's Modified Eagle Medium (DMEM; Gibco, USA; C11995500BT) containing 10% fetal bovine serum (FBS; BioSun, China; BS‐0003‐500) and 1% penicillin‐streptomycin (Gibco, USA; 15140‐122). The culture flasks or plates were placed in a humidified incubator (Thermo Fisher, USA) with 5% CO₂.

### 5.2 Cell Viability Assay

PLB (MCE, China; HY‐N1497) was dissolved in DMSO (Sigma Aldrich, USA; D2650). HCC cells (1 × 10⁴ cells/well in a 96‐well plate) were cultured with 100 µL of fresh medium containing 1 µM CuCl₂ and diverse concentrations of PLB for 24 h. Cell viability was then assessed using the Cell Counting Kit‐8 (CCK‐8; Beyotime, China; C0039). Ten microliters of CCK‐8 solution was added to each well, followed by incubation at 37°C for 1 h. Absorbance was measured at 450 nm using a microplate reader (Tecan, Switzerland; Sunrise). Based on the determined half‐maximal inhibitory concentrations (IC50) for both cell lines, 6 µM PLB was selected for subsequent experiments. To rescue HCC cells from PLB‐induced phenotypes, 20 µM TTM (Sigma Aldrich, USA; 32344) was added 12 h before the addition of PLB.

### 5.3 Colony Formation, Transwell, Wound‐Healing, and Cell Cycle Analysis Assays

In a colony formation assay, cells (1 × 10⁴/well) were plated in six‐well plates. The following day, attached cells were treated with DMSO, PLB, or PLB + TTM for 12 h. Every 3 days, the medium was replaced with fresh culture medium to allow colony formation. Two weeks later, fixed colonies were stained with crystal violet solution (Beyotime, China; C0121), imaged using a microscope (Olympus, Japan; IX71), and counted with ImageJ software (National Institutes of Health, USA).

In the Transwell assay, cells (5 × 10⁴) were treated with DMSO, PLB, or PLB + TTM for 12 h, then seeded in the upper chamber (8‐µm pore size; Corning, USA; 3470) with serum‐free DMEM. Serum‐containing medium was added to the lower chamber. After 48 h of incubation at 37°C, cells were fixed and stained with crystal violet solution. Stained cells were imaged and counted.

In the wound‐healing assay, cells were cultured in 24‐well plates to 100% confluence. Then, the wound gaps were generated using a sterile pipette tip (200 µL), and detached cells were washed with serum‐free medium. The remaining cells were treated with DMSO, PLB, or PLB + TTM for 12 h, followed by medium replacement with DMEM containing 2% FBS. After 48 h, images of the wound gap were taken, and the healing area was quantified using ImageJ software.

In the cell cycle analysis, cells (5 × 10⁵/well in a six‐well plate) were treated with PLB or PLB + TTM for 12 h. After treatment, cells were harvested, fixed in 70% ice‐cold ethanol overnight at 4°C, washed with phosphate buffer saline (PBS; Biosharp, China; BL302A), and stained with propidium iodide/RNase Staining Buffer (BD Biosciences, USA; 550825) for 30 min in the dark. Cell cycle distribution was analyzed by flow cytometry (SONY, Japan; MA900) and processed using FlowJo 10.1 software (Tree Star, Inc., USA).

### 5.4 Copper Assay

Intracellular copper levels were measured using a copper colorimetric assay kit (NJJCBIO, China; E010‐1‐1) following the manufacturer's protocol. Absorbance was recorded at 600 nm and normalized to protein concentration.

### 5.5 Western Blotting

Cells were lysed in a RIPA lysis buffer (Beyotime, China; P0013B) supplemented with protease and phosphatase inhibitors (Beyotime, China; P1050). Proteins were extracted and denatured. Equal amounts of protein (20 µg) were separated on a 10% SDS‐PAGE gel (Beyotime, China; P0455M) and transferred to polyvinylidene fluoride (PVDF) membranes (Millipore, Germany; IPVH00010). Membranes were blocked with 5% bovine serum albumin (BSA) in TBST to prevent nonspecific binding, then incubated with primary antibodies. Primary antibodies included anti‐β‐tubulin (Affinity Biosciences, China; T0023), anti‐LIAS (Proteintech, China; 11577‐1‐ap), and anti‐Histone H3 (Beyotime, China; AF0009). Additional antibodies used were anti‐DLAT (4A4‐B6‐C10) and anti‐DNMT1 (D63A6) from Cell Signaling Technology (USA), as well as anti‐FDX1 (ab108257), anti‐SLC31A1 (ab129067), and anti‐ATP7B (ab131208) from Abcam (UK). Protein bands were visualized using corresponding horseradish peroxidase (HRP)‐conjugated secondary antibodies and an enhanced chemiluminescence (ECL) system (Beyotime, China; P0018FM), and semi‐quantitative analysis was conducted with Image Lab software (Bio‐Rad, USA).

### 5.6 Total RNA Isolation, Reverse Transcription, and Quantitative Polymerase Chain Reaction

Total RNA was extracted from HCC cells and mouse tumor tissues using an RNA extraction kit (Promega, USA; LS1040) following the manufacturer's protocol. Equal amounts of RNA were reverse‐transcribed into first‐strand cDNA using a reverse transcription kit (Promega, USA; A2800). Quantitative polymerase chain reaction (qPCR) was performed to assess mRNA expression using the Go Taq qPCR and RT‐qPCR Systems (Promega, USA; A6001). For miRNA analysis, cDNA synthesis and qPCR were carried out using the miRcute Plus miRNA First‐Strand cDNA Kit (KR211‐02) and qPCR Kit (FP411‐02) from TIANGEN (China). Primer sequences are listed in Table S1. β‐actin served as the internal control for mRNA quantification, while U6 was used for normalization of miRNA expression. Specificity of amplification was confirmed by melting curve analysis. Relative expression levels of mRNAs and miRNAs were calculated using the 2^(−ΔΔCt)^ method.

### 5.7 Immunofluorescence

Cells were seeded onto coverslips in 24‐well plates and incubated overnight, followed by the indicated treatments. For mitochondrial labeling, cells were stained with 200 nM MitoTracker Deep Red FM (Cell Signaling Technology, USA; 8778S) for 30 min at 37°C. After staining, cells were fixed with 4% paraformaldehyde for 10 min, permeabilized using 0.25% Triton X‐100 for 10 min, and blocked with 3% BSA for 1 h. Subsequently, cells were incubated with anti‐DLAT antibody (1:300) at 4°C overnight. After three washes with TBST, cells were treated with the secondary antibody (Cell Signaling Technology, USA; 4408S) for 1 h at room temperature, followed by washing and mounting with a DAPI‐containing medium (Beyotime, China; P0131). Confocal images were acquired using a TCS SP8 microscope (Leica, Germany).

### 5.8 Measurements of Lipid‐ROS, Enzyme Activities, Metabolites, and MDA

To assess lipid‐ROS levels, cells were treated with DMSO, PLB, or PLB plus TTM for 24 h, followed by staining with 3 µM C11‐BODIPY 581/591 (Invitrogen, USA; D3861) in serum‐free DMEM for 30 min at 37°C. After staining, cells were washed, trypsinized, and resuspended in PBS. The fluorescence intensity was measured using a flow cytometer, and the MFI was calculated to evaluate lipid‐ROS levels.

Levels of GSH (NJJCBIO, China; A006‐2‐1), LPO (NJJCBIO, China; A106‐1‐1), SOD (NJJCBIO, China; A001‐1‐1), CAT (NJJCBIO, China; A007‐1‐1), LA (NJJCBIO, China; A019‐2‐1), PA (NJJCBIO, China; A081‐1‐1), and MDA (Beyotime, China; S0131S) in HCC cells were measured using commercial assay kits. Data were collected using a microplate reader and normalized to protein concentration.

### 5.9 Transfection of miRNAs or Plasmids

MiR‐302a‐3p mimic, inhibitor, and negative control (NC) mimic were generated by GenePharma (China), while expression plasmids for ATP7B, DNMT1, and empty vector were constructed by GeneChem (China). Cells were transfected with mimics (50 nM), inhibitor (100 nM), or plasmids (1–2 µg) using jetPRIME reagent (Polyplus, France; 10100046). The expression levels of miR‐302a‐3p or proteins were examined 48–72 h after transfection, and the cells were used for subsequent experiments.

### 5.10 Luciferase Reporter Assay

To evaluate potential binding between miR‐302a‐3p and ATP7B 3’UTR, wild‐type sequences containing the binding sites of miR‐302a‐3p in ATP7B 3’UTR or mutant sequences were inserted into the vector PDS131 to construct wild‐type and mutant luciferase reporter plasmids. Subsequently, cells were seeded into 24‐well plates at a density of 1 × 10^4^ cells per well. The luciferase reporter (0.5 µg) and hsa‐miR‐302a‐3p mimics (50 nM) or negative control miR‐NC (50 nM) were co‐transfected into HEK‐293 cells. After 48 h, luciferase activity was detected using the dual‐luciferase reporter assay kit (Beyotime, China; RG027).

### 5.11 Methylation‐Specific PCR

The genomic DNA of HCC cells was extracted using the Genomic DNA Mini Extraction Kit (Beyotime, China; D0063). The unmethylated DNA underwent bisulfite conversion using EZ DNA Methylation‐Gold Kit (Zymo Research, USA; D5005). The primers specific for the unmethylated miR‑302a‐3p promoter are shown in Table S1. The protocol for methylation‐specific PCR was as follows: 95°C for 5 min; followed by 40 cycles at 95°C for 30 s, 55°C for 30 s, and 72°C for 30 s; and extension at 72°C for 10 min. PCR products were separated in 1% agarose gels.

### 5.12 Animal Experiments

Male BALB/c nude mice (5–6 weeks old) were obtained from GemPharmatech Co., Ltd (China). A previously reported tolerated dose of PLB (2 mg/kg/day) was administered intraperitoneally five times per week. After 2 weeks of treatment, the safety of PLB was confirmed by blood parameter analysis and histological examination of major organs. To establish subcutaneous xenografts, Huh7 cells (5 × 10⁶ cells/mouse) were injected into the right flank of each mouse. Mice (*n* = 5 per group) were then treated intraperitoneally with DMSO, PLB (2 mg/kg/day), or PLB (2 mg/kg/day) combined with TTM (10 mg/kg/day), five times per week. After 2 weeks, mice were sacrificed, and tumors were excised, weighed, and collected for further analysis. Tumor volume was calculated using the formula: volume (mm^3^) = (width^2^ × length) / 2.

The xenograft tumors were embedded in paraffin. Five‐micrometre‐thick sections were prepared, placed on glass slides, stained with H&E (Servicebio, China; G1004), or processed for immunofluorescence analysis. The slides underwent deparaffinization, rehydration, antigen retrieval, blocking, and incubation at 4°C overnight with the mixed primary antibodies against ATP7B (Affinity Biosciences, China; AF0410) and Ki‐67 (Servicebio, China; GB111499) or the mixture of DLAT (Cell Signaling Technology, USA; 4A4‐B6‐C10) and TOMM20 (Abcam, UK; EPR15581‐54) antibodies. The next day, sections were incubated with secondary antibodies at room temperature for 1 h, followed by counterstaining DAPI for nucleus and quenching tissue autofluorescence. Finally, the slides were sealed with coverslips and imaged using the Pannoramic MIDI (3DHISTECH, Hungary). The protein, mRNA, and miRNA levels in tumor samples were also analyzed by Western blotting and qPCR.

The Animal Ethics Committee of Shanghai Medical College (Fudan University) approved the animal experimental protocols (202312017S, December 23, 2023), which followed the guidelines of the Institutional Animal Care and Use Committee (Fudan University).

### 5.13 Prognostic Analyses Using Clinical Samples

Paraffin‐embedded tumor tissue samples were collected from 30 HCC patients, with recurrence information recorded. Immunohistochemical staining for ATP7B was performed on tissue slides, and the final staining results were assessed independently using ImageJ software. The staining intensity was categorized into four grades: negative (0), weak (0–1), moderate (1–1.5), and strong (1.5–3). The ATP7B score of each sample was obtained by multiplying the staining intensity grade by the area percentage of positive cells (0%–100%). Cases were classified as low‐level or high‐level groups using the optimal ATP7B threshold. The prognostic value of ATP7B level was evaluated by Kaplan–Meier analyses. Univariate COX regression analysis was performed to examine if ATP7B score and ATP7B level were risk factors for recurrence. The use of human subjects (B2019‐059R, March 22, 2019) was approved by the Ethics Committee of Zhongshan Hospital (Fudan University), and written informed consent was obtained from all subjects.

### 5.14 Statistical Analysis

Statistical analyses were conducted using Prism software (GraphPad, USA; version 9.5). Data are presented as mean ± standard deviation (SD). Student's *t*‐test was applied for comparisons between two groups, while multiple‐group comparisons were made using one‐way ANOVA. Statistical significance was defined as *p* < 0.05.

## Author Contributions

Chuyu Wang: conceptualization, validation, formal analysis, investigation, data curation, writing—original draft, visualization. Hao Wang: validation, investigation, data curation, supervision, writing—original draft, writing—review and editing. Chong Wang: resources, formal analysis, writing—review and editing. Tongtong Tian: project administration, data curation. Anli Jin: methodology, investigation. Yu Liu: methodology, investigation. Ran Huo: methodology. Te Liu: conceptualization, supervision. Baishen Pan: supervision. Wei Guo: supervision, funding acquisition. Wenjing Yang: supervision, project administration. Beili Wang: conceptualization, supervision, writing—review and editing. All authors have read and approved the final manuscript.

## Ethics Statement

The Animal Ethics Committee of Shanghai Medical College (Fudan University) approved the animal experimental protocols (202312017S, December 23, 2023) and the Ethics Committee of Zhongshan Hospital (Fudan University) approved the clinical study (B2019‐059R, March 22, 2019). Written informed consent was obtained from all subjects.

## Conflicts of Interest

The authors declare no conflicts of interest.

## Supporting information



Supporting Information

## Data Availability

All data are available within the main text and Supporting Information.
