# Supplementary material for: Plumbagin Triggers Cuproptosis in Hepatocellular Carcinoma (HCC) via the DNA‐Methyltransferase 1 (DNMT1)/microRNA‐302a‐3p (miR‐302a‐3p)/ATPase Copper Transporting Beta (ATP7B) Axis
Source: MedComm (2020). 2025 Aug 3;6(8):e70312. doi: 10.1002/mco2.70312 (PMC12318818; doi:10.1002/mco2.70312)
Supplement: Supplementary file 1 — Supporting Information [file MCO2-6-e70312-s001.docx]

**Title**

Plumbagin triggers cuproptosis in hepatocellular carcinoma (HCC) via the DNA-methyltransferase 1 (DNMT1)/microRNA-302a-3p (miR-302a-3p)/ATPase copper transporting beta (ATP7B) axis

**Running title**

Plumbagin (PLB) triggers cuproptosis in hepatocellular carcinoma (HCC)

**Author names**

Chuyu Wang^1,#^, Hao Wang^1,#^, Chong Wang^1,#^, Tongtong Tian^1^, Anli Jin^1^, Yu Liu^1^, Ran Huo^1^, Te Liu^5^, Baishen Pan^1^, Wei Guo^1,2,3,4,*^,Wenjing Yang^1,*^, Beili Wang^1,*^

**Affiliations**

^1^ Department of Laboratory Medicine, Zhongshan Hospital, Fudan University, Shanghai, China

^2^ Department of Laboratory Medicine, Shanghai Geriatric Medical Center, Shanghai, China

^3^ Department of Laboratory Medicine, Wusong Central Hospital, Baoshan District, Shanghai, China

^4^ Department of Laboratory Medicine, Xiamen Branch, Zhongshan Hospital, Fudan University, Xiamen, China

^5^ Shanghai Geriatric Institute of Chinese Medicine, Shanghai University of Traditional Chinese Medicine, Shanghai, China.

^*^ Correspondence

Beili Wang, Department of Laboratory Medicine, Zhongshan Hospital, Fudan University, Shanghai, China.

Email: wang.beili1@zs-hospital.sh.cn

Wenjing Yang, Department of Laboratory Medicine, Zhongshan Hospital, Fudan University, Shanghai, China.

E-mail: yang.wenjing@zs-hospital.sh.cn

Wei Guo, Department of Laboratory Medicine, Zhongshan Hospital, Fudan University, Shanghai, China.

Email: guo.wei@zs-hospital.sh.cn & zs-guowei@hotmail.com

^#^ Chuyu Wang, Hao Wang, and Chong Wang contributed equally to this work.

**Acknowledgements**

We thank generous support from the Liver Cancer Institute, Zhongshan Hospital, Fudan University. This work was supported by the National Natural Science Foundation of China (82172348, 82102483, 82202636), Baoshan District Health Commission Key Subject Construction Project (BSZK-2023-A18), the Constructing Project of Clinical Key Disciplines in Shanghai (SHSLCZDZK03302), Shanghai Sailing Program (22YF1406300) and Scientific Research Fund by Zhongshan Hospital (418).

**A**

**B**

**Supplementary Figure 1.** PLB induces cuproptosis-dependent cell death

(A) Inhibitory effects of PLB (6 μM) on Huh7 and PLC cells in the presence of copper chelator (20 μM TTM), ferroptosis inhibitors (2 μM Fer-1 [HY-100579-5mg, MCE, China] and 100μM DFO [HY-D0903, MCE, China]), necroptosis inhibitor (2 μM Nec-1 [HY-15760, MCE, China]) or apoptosis inhibitors (5 μM Z-VAD-FMK [HY-16658B, MCE ,China] and 30 μM Boc-D-FMK [HY-13229, MCE ,China]) were analyzed via the CCK-8 assay. N = 3; ^∗^*P* < 0.05, ^∗∗^*P* < 0.01, and ^∗∗∗^*P* < 0.001 compared to the PLB group; ^#^*P* < 0.05 compared to the PLB+TTM group; ns, not significant. (B) Time-course effects of PLB and/or TTM on the viability of HCC cells. Relative cell viability vs the corresponding DMSO group at the same time point was calculated. N = 5; ^∗∗∗^*P* < 0.001; ns, not significant.

PLC

DMSO

PLB

PLB+TTM


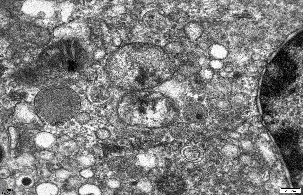

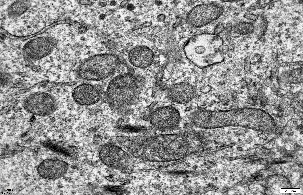

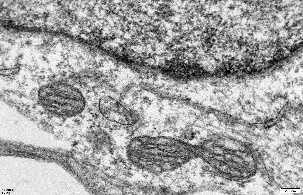


Huh7


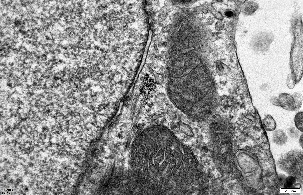

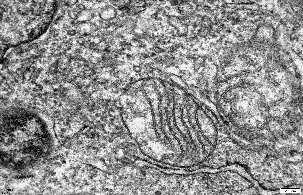

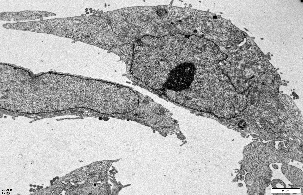

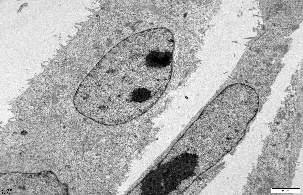

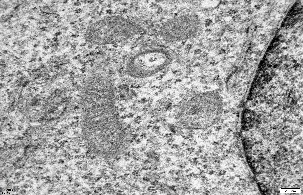

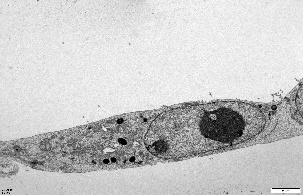

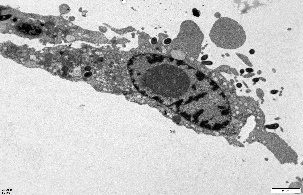

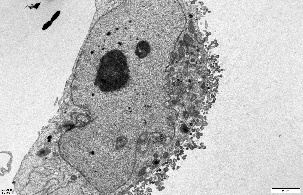

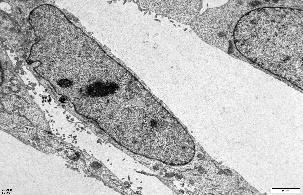


DMSO

PLB

PLB+TTM

**Supplementary Figure 2.** PLB promotes mitochondrial damage

Transmission electron microscopy results showed that the mitochondria were damaged in PLB-treated HCC cells. The red arrows indicate mitochondria. N = 3; ^∗^*P* < 0.05, ^∗∗^*P* < 0.01, and ^∗∗∗^*P* < 0.001.

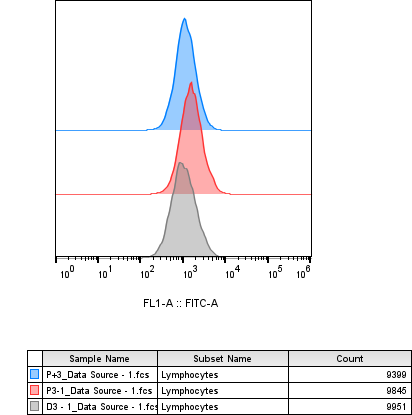


C11-BODIPYTM581/591(FL1)

ATP7B-OE

+PLB

PLB

DMSO

1296

1815

1260


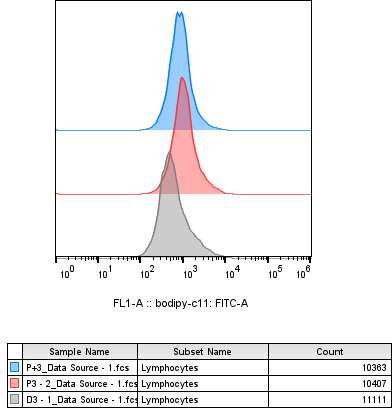


C11-BODIPYTM581/591(FL1)

ATP7B-OE

+PLB

PLB

DMSO

1026

1330

893

**A**

**B**

**C**

**D**

**E**

**Supplementary Figure 3.** ATP7B mediates PLB-induced oxidative stress

(A) The lipid-ROS in ATP7B-OE HCC cells. (B-D) The content of intracellular GSH, LPO and MDA of ATP7B-OE HCC cells after PLB treatment. (E) The hydroxyl radical (HO·) scavenging activity of ATP7B-OE HCC cells after PLB treatment. N = 3; ^∗^*P* < 0.05, ^∗∗^*P* < 0.01, and ^∗∗∗^*P* < 0.001.


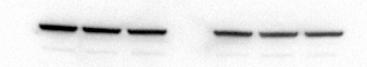

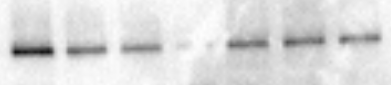


β-tubulin

ATP7B


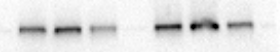

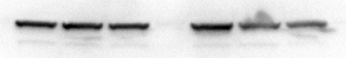


PLC

Huh7

miR-302d-3p

- + - +

1.0

1.0

1.3

0.6

150kDa

55kDa

**A**

**B**

**C**


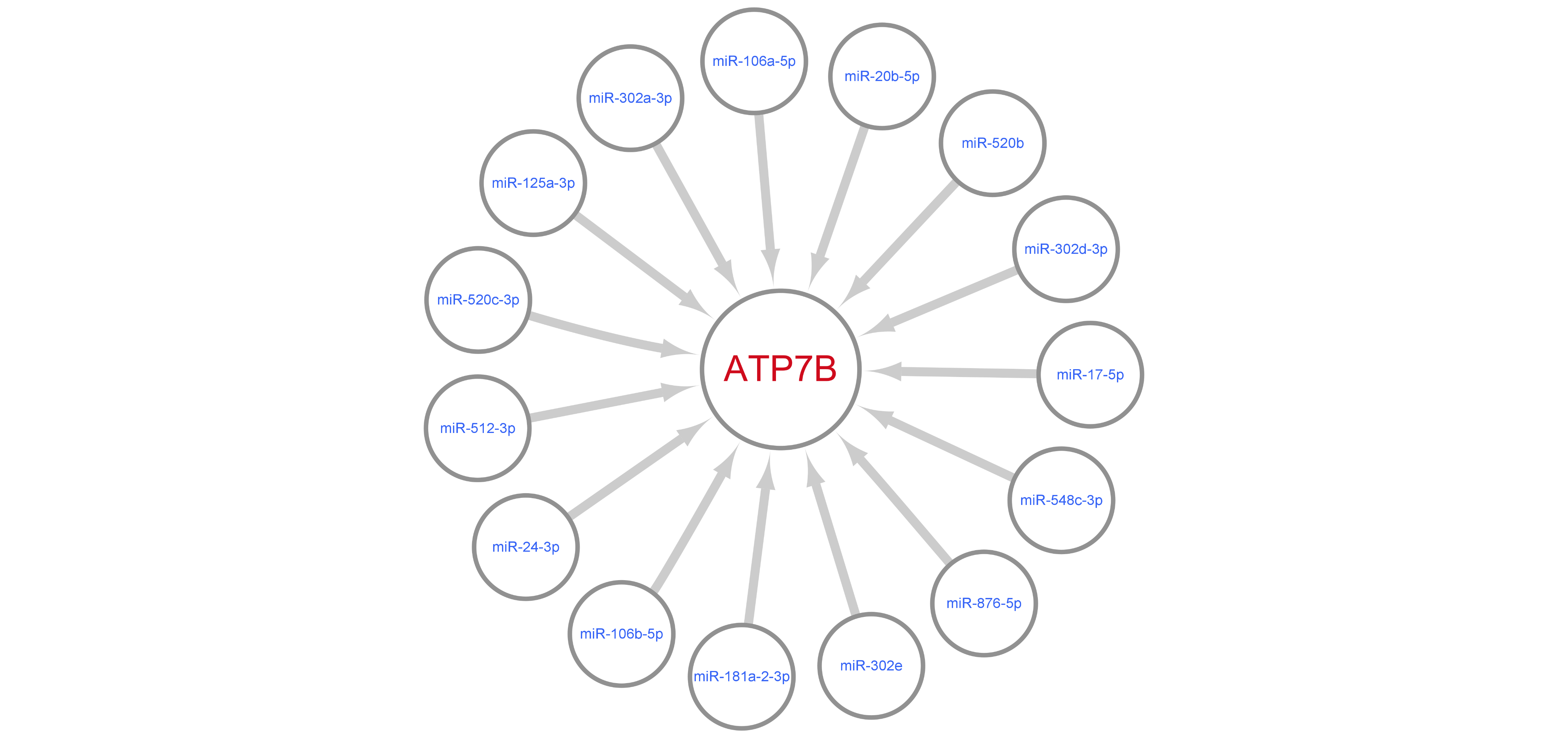


**Supplementary Figure 4.** PLB alters the expression levels of the potential ATP7B-regulating miRNAs

(A) Predicted miRNAs that may regulate ATP7B. (B) The expression levels of 15 predicted miRNAs in HCC cells after PLB treatment. The miRNAs upregulated in both cell lines were circled. N = 3; ^∗^*P* < 0.05, ^∗∗^*P* < 0.01, and ^∗∗∗^*P* < 0.001; ns, not significant. (C) Western blotting analysis of ATP7B in HCC cells transfected with miR-NC or miR-302d-3p mimic.

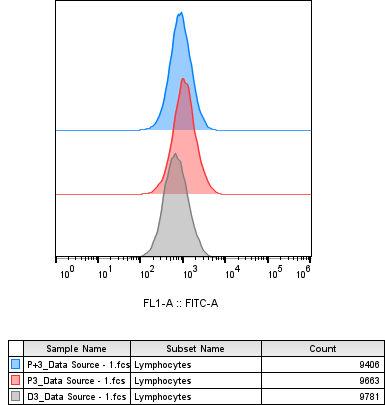


C11-BODIPYTM581/591(FL1)

PLB+

miR-302a-3p

inhibitor

PLB

DMSO

998

1252

830

**A**

**B**

**C**

**D**

**E**


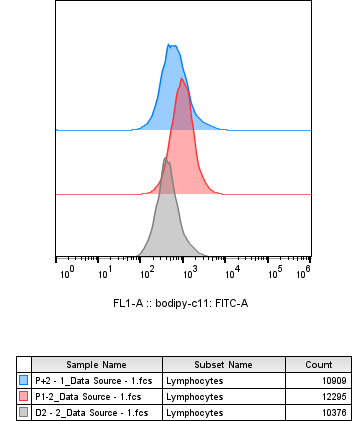


C11-BODIPYTM581/591(FL1)

PLB+

miR-302a-3p

inhibitor

PLB

DMSO

874

1123

578

**Supplementary Figure 5.** miR-302a-3p mediates PLB-induced oxidative stress

(A) The lipid-ROS in miR-302a-3p-inhibiting HCC cells. (B-D) The content of intracellular GSH, LPO and MDA of miR-302a-3p-inhibiting HCC cells after PLB treatment. (E) The hydroxyl radical (HO·) scavenging activity of miR-302a-3p-inhibiting HCC cells after PLB treated. N = 3; ^∗^*P* < 0.05, ^∗∗^*P* < 0.01, and ^∗∗∗^*P* < 0.001.

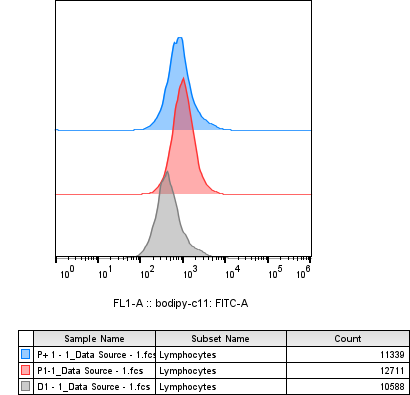


C11-BODIPYTM581/591(FL1)

PLB+

DNMT1-OE

PLB

DMSO

1079

1175

664

C11-BODIPYTM581/591(FL1)


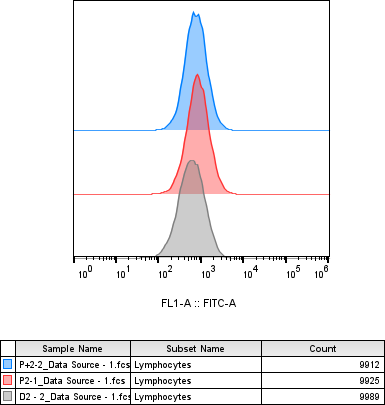


PLB+

DNMT1-OE

PLB

DMSO

867

972

727

**A**

**B**

**C**

**D**

**E**

**F**

**
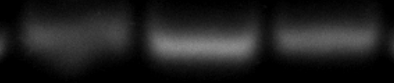

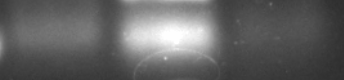
**

PLB

DNMT1-OE

-

+

+

-

-

+

-

+

+

-

-

+

Huh7

PLC

1.0

1.5

1.3

1.0

3.7

0.7

Unmethylated

**Supplementary Figure 6.** DNMT1 mediates PLB-induced oxidative stress

(A) The lipid-ROS in DNMT1-OE HCC cells. N = 3. (B-D) The content of intracellular GSH, LPO and MDA of DNMT1-OE HCC cells after PLB treatment. N = 3. (E) The hydroxyl radical (HO·) scavenging activity of DNMT1-OE HCC cells after PLB treated. N = 3. (F) The unmethylation status of the miR-302a-3p promoter in DNMT1-OE HCC cells. ^∗^*P* < 0.05, ^∗∗^*P* < 0.01, and ^∗∗∗^*P* < 0.001.


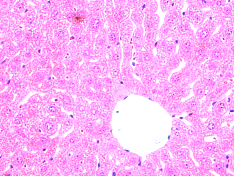

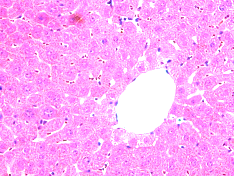

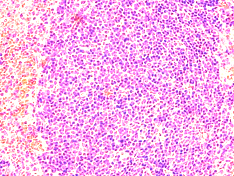

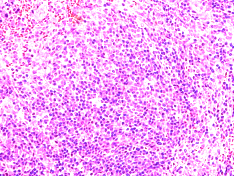

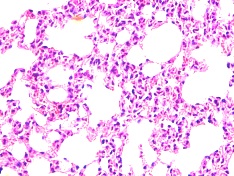

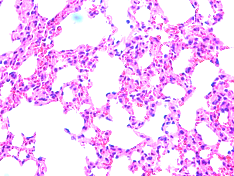

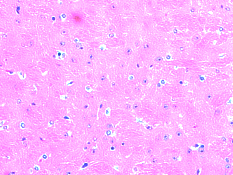

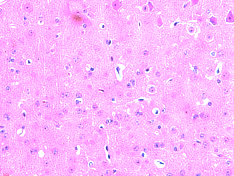

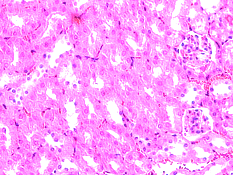

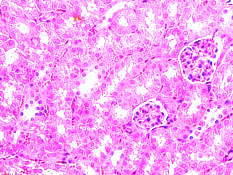

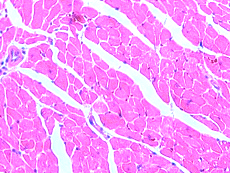

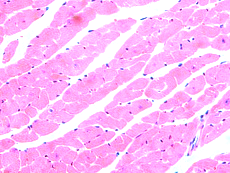


**A**

**E**

**Liver**

**Spleen**

**lung**

**Brain**

**Kidney**

**Heart**

200μm

**B**

**C**

**D**

**F**

**G**

**I**

**H**

**J**

**K**

**L**

**N**

**O**

**P**

**Q**

**M**

**Supplementary Figure 7.** Effects of PLB on parameters of blood and morphology of major organs

(A-P) WBC, RBC, HGB, PLT, TBIL, DBIL, ALT, AST, GGT, CHE, CK, ALBP, ECRE, TP, UA and UN in PLB-treated mice. N = 5; ns, not significant. (Q) Representative images of H&E-stained liver, spleen, lung, brain, kidney and heart samples. Scale bars: 200 μm.

**A**

**B**

**D**

**E**

**F**

**G**

**H**

**C**


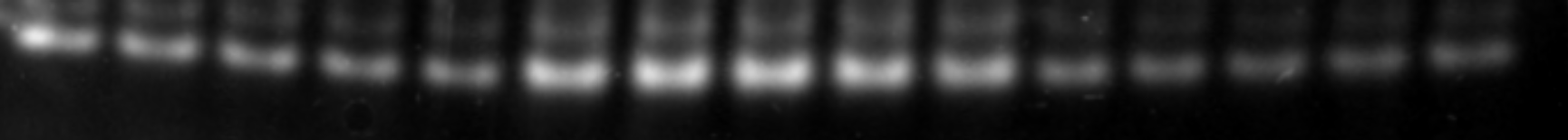


**I**

**J**

DMSO

PLB

Unmethylated

PLB+TTM

**Supplementary Figure 8.** PLB increases oxidative stress *in vivo*

(A-G) The content of GSH, MDA, LPO, SOD, CAT, LA and PA in tumor tissues. (H) The hydroxyl radical (HO·) scavenging activity. (I) The unmethylation status of the miR-302a-3p promoter. (J) The expression levels of ATP7B mRNA. N = 5; ^∗^*P* < 0.05, ^∗∗^*P* < 0.01, and ^∗∗∗^*P* < 0.001; ns, not significant.


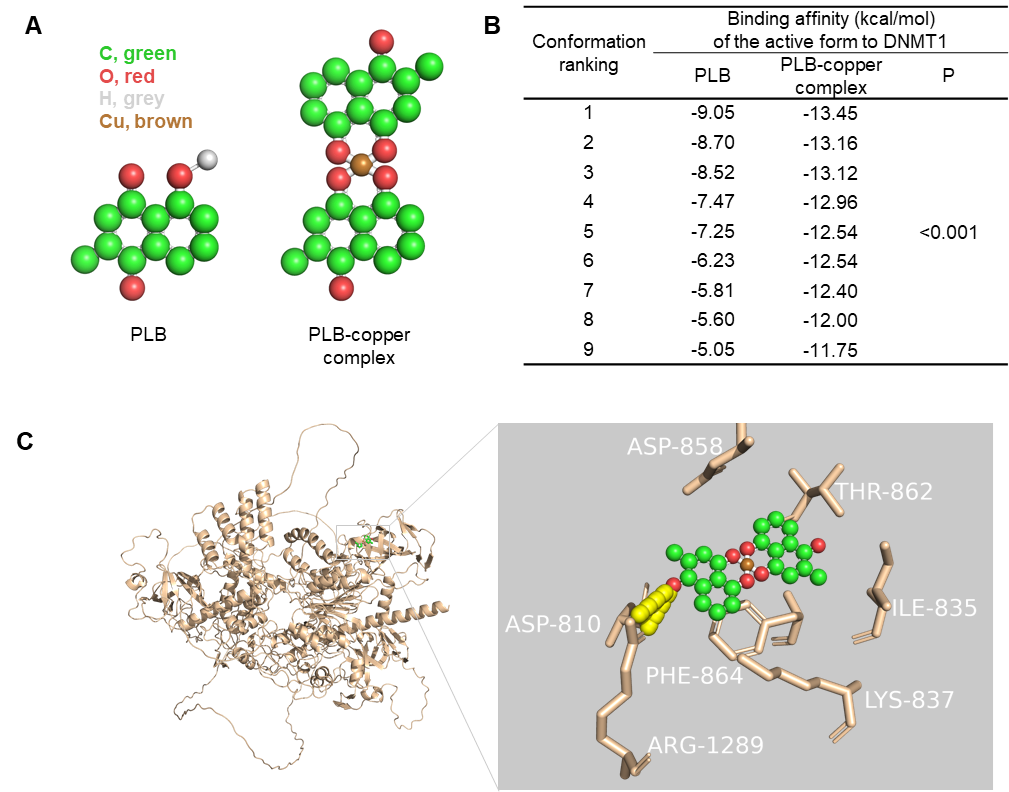


**Supplementary Figure 9.** Molecular docking of DNMT1 and the active form of PLB

(A) The ball-and-stick models of PLB and the PLB-copper complex. (B) Binding affinity of PLB or the PLB-copper complex to DNMT1. The molecular docking was performed using GNINA tool on Neurosnap webserver (https://neurosnap.ai/service/GNINA) and the binding affinities of top ranked conformation were listed. The binding affinity of the PLB-copper complex was significantly stronger than that of PLB. (C) The docking structure (left) and site (right) with the strongest binding affinity. The active form here is the PLB-copper complex.

**Supplementary Table 1.** PCR primers

| Gene Name | Forward primers (5'→3') | Reverse primers (5'→3') |
| --- | --- | --- |
| ATP7B | ATATTGAGCGGTTACAAAGCACT | ATATTGAGCGGTTACAAAGCACT |
| SLC31A1 | AAGGACTCAAGATAGCCCGAG | TGGGACAGGCATGGAATTGTA |
| ACTB | CATGTACGTTGCTATCCAGGC | CTCCTTAATGTCACGCACGAT |
| miR-548c-3p | CAAAAATCTCAATTACTTTTGC | TIANGEN (FP411-02) Reverse Primer |
| miR-181a-2-3p | ACCACTGACCGTTGACTGTACC | TIANGEN (FP411-02) Reverse Primer |
| miR-24-3p | TGGCTCAGTTCAGCAGGAACAG | TIANGEN (FP411-02) Reverse Primer |
| miR-520c-3p | AAAGTGCTTCCTTTTAGAGGGT | TIANGEN (FP411-02) Reverse Primer |
| miR-520b | CCCTCTACAGGGAAGCGCTTTCTGTTGTCTGAAAGAAAAGAAAGTGCTTCCTTTTAGAGGG | TIANGEN (FP411-02) Reverse Primer |
| miR-302d-3p | TAAGTGCTTCCATGTTTGAGTGT | TIANGEN (FP411-02) Reverse Primer |
| miR-302e | TAAGTGCTTCCATGCTT | TIANGEN (FP411-02) Reverse Primer |
| miR-302a-3p | TAAGTGCTTCCATGTTTTGGTGA | TIANGEN (FP411-02) Reverse Primer |
| miR-106a-5p | TAAAGTGCTGACAGTGCAGAT | TIANGEN (FP411-02) Reverse Primer |
| miR-125a-3p | ACAGGTGAGGTTCTTGGGAGCC | TIANGEN (FP411-02) Reverse Primer |
| miR-876a-5p | TGGATTTCTTTGTGAATCACCA | TIANGEN (FP411-02) Reverse Primer |
| miR-20b-5p | CAAAGTGCTCATAGTGCAGGTAG | TIANGEN (FP411-02) Reverse Primer |
| miR-512-3p | TGATGGCGAAGCGAGTGAAG | TIANGEN (FP411-02) Reverse Primer |
| miR-106a-5p | AAAAGTGCTTACAGTGCAGGTAG | TIANGEN (FP411-02) Reverse Primer |
| miR-17-5p | CAAAGTGCTTACAGTGCAGGTAG | TIANGEN (FP411-02) Reverse Primer |
| U6 | CTCGCTTCGGCAGCACA | AACGCTTCACGAATTTGCGT |
